# Valorization of monovarietal *Nostrana di Brisighella* extra virgin olive oils: focus on bioactive compounds

**DOI:** 10.3389/fnut.2024.1353832

**Published:** 2024-04-04

**Authors:** Enrico Casadei, Enrico Valli, Alessandra Bendini, Sara Barbieri, Rosalba Tucci, Federico Ferioli, Tullia Gallina Toschi

**Affiliations:** ^1^Department of Agricultural and Food Science, Alma Mater Studiorum – Università di Bologna, Bologna, Italy; ^2^Interdepartmental Centre for Industrial Agrofood Research, Alma Mater Studiorum – Università di Bologna, Cesena, Italy

**Keywords:** extra virgin olive oil, sensory analysis, organic farming, integrated pest management, olive ripening degree, volatile compounds, phenolic compounds, health claim

## Abstract

A “green breakthough” at the table due to consumer demand for healthy and sustainable foods, which aligns with the typical Mediterranean diet, has recently led to an increase in the consumption of products such as extra virgin olive oil. In fact, Italian olive cultivation, which contributes an average of 15% of world production, has seen the production of extra virgin olive oil with a value of exports that have doubled in the last 20 years. In this context, the olive oil sector of the Emilia-Romagna region (Italy), and in particular the PDO Brisighella, could achieve greater success with consumers by proposing a product obtained through sustainable agriculture that enhances the content of bioactive compounds. For these reasons, in this study, different agronomic variables are investigated in order to optimize the presence of bioactive components in extra virgin olive oil made from monovarietal *Nostrana di Brisighella*, namely phenolic and positive volatile compounds, thus naturally enriching this product both from health and sensory points of view. The study focuses on the volatile and phenolic fractions (derivatives of hydroxytyrosol and tyrosol) of olive oil and the positive sensory attributes (fruity, bitter and pungent) that are known to be associated with these molecules. The phenolic content is of particular interest due to the potential to support health claims. Extra virgin olive oil samples were produced from olives of the *Nostrana di Brisighella* cultivar; fruits were obtained through integrated pest management or organic farming and picked at four increasing indices of maturity, corresponding to four successive weeks of harvesting. These agronomic variables influenced the compositional and sensory characteristics of the extra virgin olive oils assessed, highlighting differences that likely derive from the effect of the agronomic system used, i.e., integrated pest management or organic farming.

## 1 Introduction

Olive oil has always been known for its excellent nutritional and health properties. In fact, as one of the main components of the Mediterranean diet (1), many epidemiological studies have indicated that its intake is inversely proportional to the development of several human diseases (2). Virgin olive oils are characterized by the presence of numerous minor compounds responsible for its flavor (e.g., volatile and phenolic compounds) that can be influenced by different intrinsic (e.g., variety of olive fruits and maturity index) and extrinsic factors such as pedo-climatic conditions, agronomic techniques, harvesting methods, and oil storage modalities (3). This variability greatly influences the organoleptic and nutraceutical differences of extra virgin olive oils (EVOOs) (4). Therefore, the characterization of EVOOs is crucial to utilize their potential beneficial effects, their organoleptic properties and, not least, the related economic value. It is estimated that olive oil production in the European Union (EU) could reach 2.5 million tons by 2031, a 22% increase compared to 2020, due to the use of olive tree varieties that are more resistant and produce higher yields (5). Furthermore, regarding consumption in the EU, growth is expected to be driven by non-producing countries, which represented 21% of consumption in 2020 and expected to account for 32% in 2031, highlighting the increased demand of a high quality product while respecting the environment (5, 6). Especially in the Mediterranean basin and neighboring zones, where 95% of the world’s olive cultivation area is located, farming and production of olive oil is an important economic activity and a substantial source of employment (7). However, the environmental impacts related to olive tree farming and subsequent virgin olive oil extraction and management of wastes are remarkable (8). For this reason, strategies to improve sustainability, productivity, and quality are urgently needed in the olive oil sector (6). Although the management and disposal of oil mill by-products and waste represent a relevant environmental problem in all olive oil producers, in Mediterranean countries particular attention must also be paid to the management of agronomic practices in olive groves (9). Climate change also has a substantial impact on current cultivation techniques, prompting the need to adopt strategies aimed at controlling and/or mitigating its effects (10), aiming to guarantee a balance between sustainability and profitability, considering protection of the environment and avoiding the overexploitation of natural resources.

A renewal of the sustainability elements of the olive oil supply chain is therefore necessary to improve competitiveness in an increasingly globalized market to meet the needs of the modern consumer. Today, food consumption is not only linked to physiological needs but must also provide ethical, environmental, and health benefits (11). This trend translates into demand for specific agri-food products to which all stakeholders in the supply chain must promptly respond. As one of the possible consequences of this trend, the total organic olive-growing area in Italy increased by 10.5% in 2022 compared to the previous year (12). The use of sustainable agronomic practices (e.g., organic, or integrated pest management) contributes to preserving biodiversity and environmental balance, ensuring the production of high-quality and more environmental-friendly olive oils (13). Furthermore, the sustainability of EVOO is also related to local production. The natural and cultural environment plays a key role in the qualitative differentiation of local products, becoming a component of differentiation and increasing the value of the entire olive oil supply chain (14). Italy is the country with the most olive biodiversity with more than 500 varieties cultivated, from which EVOOs with unique and distinctive sensory properties are obtained, thus establishing a deep link between quality, local varieties, and the territory (15). According to data from the Italian national statistical institute (ISTAT), Italy stands out in Europe with the largest number of certified EVOOs: 42 Protected Designation of Origin (PDO) and 8 Protected Geographical Indication (PGI). Geographical indications (GIs) represent opportunities for territories, guaranteeing their development and valorization with positive repercussions on exports (12). The first designation of origin for EVOO registered in Italy since 1996 was the Brisighella PDO, obtained milling at least 90% of drupes of the autochthonous *Nostrana di Brisighella* cultivar. Therefore, the characteristics of this product intrinsically reflect those of *Nostrana di Brisighella.* Recently, an amendment approved in 2016 by the EU made minor modifications to the original version of the product specification of Brisighella PDO (16). One of the changes concerned the possibility of including the health claim “*olive oil polyphenols contribute to the protection of blood lipids from oxidative stress*,” as established in the EU Reg. 432/2012, on the olive oil label (17). The need for this modification arises from a first phase of zoning of the *Nostrana di Brisighella* cv. which took place in 2007, where it was noted that in all the EVOOs sampled from the sites examined, the total content of phenolic compounds was sufficient to allow the use of the health claim on the label.

Adopting an integrated approach that considers the different production stages of the olive oil supply chain is a winning strategy for obtaining high-quality, differentiated and enhanced olive oils, while creating new opportunities for sustainable growth and protect traditional and high-value local food products (e.g., PDO, PGI, organic). In light of this, to continue along the path of characterization and valorization of Brisighella EVOOs, this research focused on the study of volatile compounds, determination of phenolic content, and sensory attributes of several EVOO samples, produced by the “Cooperativa Agricola Brisighellese” (CAB) located in Emilia-Romagna region (Italy). These olive oils were obtained from 100% *Nostrana di Brisighella* olives, using different agronomic systems (organic farming *vs* integrated pest management) that were derived from olives sampled in different weeks of harvest corresponding to four distinct olive maturity indices.

## 2 Materials and methods

### 2.1 Reagents

The following standards (CAS number and purity percentage in parenthesis) were used for the analysis of volatile compounds and were purchased from Sigma-Aldrich (St. Louis, MO, USA): limonene (5989-27-5, 97%), (*E*)-2-hexenal (6728-26-3, ≥97.0%), (*Z*)-3-hexenyl acetate (3681-71-8, ≥98.0%), 1-hexanol (111-27-3, ≥99.9%), 6-methyl-5-hepten-2-one (110-93-0, ≥97.0%), acetic acid (64-19-7, ≥99.8%). 4-Methyl-2-pentanol (123-51-3, ≥95%) was used as internal standard (IS) and a mixture of *n*-alkanes from 8 to 20 carbon atoms (~40 mg/L each, in *n*-hexane) was used to calculate the LRI. Gallic acid (149-91-7, 97.5–102.5%), hydroxytyrosol (10597-60-1, ≥98.0), syringic acid (530-57-4, ≥97%), and tyrosol (501-94-0, >99.5%) were used for the quantification of phenolic compounds and purchased from Sigma-Aldrich.

### 2.2 Samples

This study, the results of which were published in a dataset (18), was carried out on eight monovarietal EVOOs produced from 100% *Nostrana di Brisighella*, a native variety of olives from orchards located in a limited area of the Emilia-Romagna region (Brisighella, Ravenna), in the north-central part of Italy. The olives used for this study came from olive groves that used two different agronomic systems (integrated pest management and organic farming). The oils were in line with what is defined by the product specification for the Brisighella DOP. The olive harvest was carried out during the 2022/23 olive oil campaign, which began on 10 October and ended on 15 November 2022. This period of time was divided into four weeks: the 1st from the 15th to the 22nd of October, the 2nd from the 23rd to the 29th of October, the 3rd from the 30th of October to the 5th of November, and finally the 4th from the 6th to 12th of November, in order to obtain olives with four different maturity index (MI) coming from integrated pest management (A) and organic farming (B). The monovarietal oil samples were produced on the same day of harvest at a local olive oil mill in Brisighella, all under the same technological conditions, and were coded as follows: 1A, 2A, 3A, 4A, 1B, 2B, 3B, 4B. The oils were bottled in 0.75 L dark glass bottles and stored at room temperature in a cold and dry place away from light until the subsequent analyses that were carried out in a short time.

### 2.3 Jaèn maturity index

At the beginning of each of the four week periods, the Jaèn MI (19) was measured on the olives coming from both integrated pest management and organic farming (see Section 2.2). The evaluation was carried out on 100 drupes, dividing them into the different classes of ripeness (0 = drupes with deep green epicarp color; 1 = drupes with yellow or yellowish green epicarp; 2 = drupes with yellowish epicarp with reddish spots; 3 = drupes with reddish or light purple epicarp; 4 = drupes with a black epicarp and totally clear flesh; 5 = drupes with black epicarp and half violet flesh; 6 = drupes with a black epicarp and purple flesh almost to the core; 7 = drupes with black epicarp and totally dark flesh).

The MI was obtained by applying the following formula:


$$IM=\left[\begin{array}{l} \left(0^{*}N_{0}\right)+\left(1^{*}\;N_{1}\right)+\left(2^{*}\;N_{2}\right)+\left(3^{*}\;N_{3}\right)\\ +\left(4^{*}\;N_{4}\right)+\left(5^{*}\;N_{5}\right)+\left(6^{*}\;N_{6}\right)+\left(7^{*}\;N_{7}\right) \end{array}\right]/100\text{,}$$

where N is the number of drupes belonging to each of the seven ripening classes.

### 2.4 Sensory analysis

Sensory analysis of the monovarietal oils was performed by the Professional Committee of DISTAL (Department of Agricultural and Food Sciences of the Alma Mater Studiorum – Università di Bologna, recognized by the Italian Ministry of Agriculture, Food Sovereignty and Forestry) according to official procedures (20–22). The optional terminology on positive sensory attributes for labeling purposes were also considered. Specifically, the term “robust” was used when the median of the positive sensory attributes (fruity, bitter, pungent) was more than 6.0; “medium,” when the median was more than 3.0 and less or equal to 6.0; “delicate,” when the median was less or equal to 3.0. Moreover, evaluation of the eventual presence and intensity of other positive attributes was carried out by the trained assessors according to the list of descriptors recognized for PDO EVOOs, as established in the IOC standards (23).

### 2.5 Extraction and determination of volatile compounds

The operating procedure for preparing the olive oil samples and the IS mixture are described in Casadei et al. (24) and Aparicio-Ruiz et al. (25) with some modifications. The sample, added with the IS mixture, was placed in a 20 mL vial that was hermetically closed with polytetrafluoroethylene septum and left for 10 min in pre-incubation at 40°C under agitation. Successively, the septum covering each vial was pierced with a needle and the solid-phase microextraction (SPME) fiber was exposed to the headspace for 40 min at 40°C. This operation was carried out with the assistance of an autosampler (AOC-5000 plus, Shimadzu, Kyoto, Japan). The SPME fiber (length 1 cm, 50/30 μm film thickness) endowed with the Stable Flex stationary phase of divinylbenzene/carboxen/polydimethylsiloxane (DVB/CAR/PDMS) (Supelco Ltd., Bellefonte, PA, USA) was then introduced into the injector port of the GC for 5 min at 250°C with the purge valve set to 1:10 ratio (split mode) and injected into a polar-phase capillary column (TG-WAXMS: length 60 m, internal diameter 0.25 mm, and coating 0.50 μm; Thermo Fisher Scientific, Waltham, MA, USA) of a GC equipped with a mass spectrometry (MS) detector (QP2010 Ultra, Shimadzu, Kyoto, Japan). The carrier gas used was helium and the oven temperature was held at 40°C for 10 min and then programmed to increase by 3°C/min to a final temperature of 200°C. A cleaning step was added at the end of the oven programmed temperature (20°C/min to 250°C for 5 min) to ensure that the column was ready for the next analysis. The MS analyzer was operated in the full-scan mode (m/z range from 30 to 250) with a scan speed of 454 (m/z)/s and electron energy of 70 eV. Furthermore, the ion source and transfer line temperature were set at 200°C and 260°C, respectively, as reported in Lozano-Castellón et al. (26). The results are expressed as the mean of three analytical replicates. The tentative identification of volatile compounds was performed by MS comparing the spectra of each analyte with the reference spectra reported in the NIST library (2008 version). Furthermore, to identify each extracted compound, the linear retention indices (LRI) are also determined and reported in Table 1. For this, the *n*-alkane mixture was injected into the GC system and the alkane retention times are used in the following equation, obtaining the LRI of each extracted analyte.


$$LRI=100\;x\;z+100\;x\;\left[\left({RT}_{\mathrm{analyte}}-{RT}_{z}\right)/\left({RT}_{z+1}-{RT}_{z}\right)\right]\text{,}$$

**Table 1:** C_5_ and C_6_ compounds formed during the LOX value pathway starting from linoleic (LA) and linolenic (LnA) acids, and minor volatile compounds reported as the sum of the chemical classes to which they belong.

C_5_, C_6_ volatile compound	LRI	OTV (mg/kg)	Concentration ± SD* ppm (mg/kg)^**^							
			1A	2A	3A	4A	1B	2B	3B	4B
(*Z*)-3-hexenal	1,219	0.0017^2^	0.59 ± 0.09	0.73 ± 0.08	0.77 ± 0.01	0.84 ± 0.03	0.56 ± 0.02	0.49 ± 0.03	0.39 ± 0.04	0.35 ± 0.00
(*E*)-2-hexenal	1,236	0.42^1^	6.96 ± 0.40	6.00 ± 0.53	6.09 ± 0.04	5.93 ± 0.52	6.91 ± 0.13	11.51 ± 0.73	20.85 ± 2.28	10.79 ± 0.37
**Ʃ C_6_/LnA-Aldehydes**			**7.55 ± 0.47** ^**c**^	**6.73 ± 0.61** ^**c**^	**6.86 ± 0.04** ^**c**^	**6.77 ± 0.54** ^**c**^	**7.47 ± 0.15** ^**c**^	**12.01 ± 0.72** ^**b**^	**21.24 ± 2.32** ^**a**^	**11.14 ± 0.37** ^**b**^
(*Z*)-3-hexen-1-ol	1,405	1.1^1^	0.84 ± 0.06	1.06 ± 0.11	1.26 ± 0.01	1.32 ± 0.11	0.89 ± 0.02	0.84 ± 0.06	1.07 ± 0.12	1.20 ± 0.05
**Ʃ C_6_/LnA-Alcohols**			**0.84 ± 0.06** ^**d**^	**1.06 ± 0.11** ^**c**^	**1.26 ± 0.01** ^**a**^	**1.32 ± 0.11** ^**a**^	**0.89 ± 0.02** ^**d**^	**0.84 ± 0.06** ^**d**^	**1.07 ± 0.12** ^**b/c**^	**1.20 ± 0.05** ^**a/b**^
(*Z*)-3-hexenyl acetate	1,333	0.20^1^	0.02 ± 0.00	0.04 ± 0.01	0.04 ± 0.00	0.04 ± 0.00	0.03 ± 0.00	0.05 ± 0.00	0.05 ± 0.01	0.07 ± 0.00
**Ʃ C**_**6**_ **/LnA-Esters**			**0.02 ± 0.00** ^**d**^	**0.04 ± 0.01** ^**c**^	**0.04 ± 0.00** ^**c**^	**0.04 ± 0.00** ^**c**^	**0.03 ± 0.00** ^**d**^	**0.05 ± 0.00** ^**b**^	**0.05 ± 0.01** ^**b**^	**0.07 ± 0.00** ^**a**^
Hexanal	1,095	0.07^1^	0.53 ± 0.03	0.56 ± 0.06	0.69 ± 0.01	0.80 ± 0.05	0.59 ± 0.03	0.65 ± 0.05	1.07 ± 0.12	0.81 ± 0.03
**Ʃ C_6_/LA-Aldehydes**			**0.53 ± 0.03** ^**e**^	**0.56 ± 0.06** ^**d/e**^	**0.69 ± 0.01** ^**c**^	**0.80 ± 0.05** ^**b**^	**0.59 ± 0.03** ^**d/e**^	**0.65 ± 0.05** ^**c/d**^	**1.07 ± 0.12** ^**a**^	**0.81 ± 0.03** ^**b**^
1-hexanol	1,368	0.4^1^	0.07 ± 0.01	0.09 ± 0.01	0.12 ± 0.00	0.13 ± 0.02	0.10 ± 0.01	0.16 ± 0.01	0.31 ± 0.03	0.28 ± 0.02
**Ʃ C**_**6**_ **/LA-Alcohols**			**0.07 ± 0.01** ^**f**^	**0.09 ± 0.01** ^**e/f**^	**0.12 ± 0.00** ^**d**^	**0.13 ± 0.02** ^**d**^	**0.10 ± 0.01** ^**e**^	**0.16 ± 0.01** ^**c**^	**0.31 ± 0.03** ^**a**^	**0.28 ± 0.02** ^**b**^
Ʃ (*E*)-2-pentenal		0.3^1^	0.32 ± 0.03	0.34 ± 0.03	0.36 ± 0.02	0.38 ± 0.03	0.30 ± 0.00	0.29 ± 0.04	0.28 ± 0.03	0.26 ± 0.01
**Ʃ C_5_/LnA-Aldehydes**			**0.32 ± 0.03** ^**b/c/d**^	**0.34 ± 0.03** ^**a/b/c**^	**0.36 ± 0.02** ^**a/b**^	**0.38 ± 0.03** ^**a**^	**0.30 ± 0.00** ^**c/d/e**^	**0.29 ± 0.04** ^**d/e**^	**0.28 ± 0.03** ^**d/e**^	**0.26 ± 0.01** ^**e**^
1-penten-3-ol	1,176	0.4^1^	1.08 ± 0.03	1.19 ± 0.08	1.15 ± 0.06	1.20 ± 0.03	0.94 ± 0.05	0.76 ± 0.09	0.56 ± 0.05	0.65 ± 0.05
(*E*)-2-penten-1-ol	1,330		0.05 ± 0.01	0.05 ± 0.00	0.04 ± 0.00	0.05 ± 0.00	0.05 ± 0.05	0.04 ± 0.00	0.03 ± 0.00	0.04 ± 0.01
(*Z*)-2-penten-1-ol	1,338	0.25^1^	0.49 ± 0.04	0.48 ± 0.04	0.43 ± 0.00	0.44 ± 0.03	0.45 ± 0.01	0.34 ± 0.02	0.34 ± 0.02	0.35 ± 0.01
**Ʃ C**_**5**_ **/LnA-Alcohols**			**1.61 ± 0.02** ^**a**^	**1.72 ± 0.12** ^**a**^	**1.62 ± 0.06** ^**a**^	**1.69 ± 0.04** ^**a**^	**1.44 ± 0.06** ^**b**^	**1.13 ± 0.11** ^**c**^	**0.93 ± 0.08** ^**d**^	**1.03 ± 0.07** ^**c/d**^
1-penten-3-one	1,035	0.05^1^; 0.00073^1^	2.61 ± 0.17	2.52 ± 0.15	2.54 ± 0.01	2.71 ± 0.23	2.43 ± 0.07	2.14 ± 0.19	2.08 ± 0.17	1.83 ± 0.13
**Ʃ C_5_/LnA-Ketones**			**2.61 ± 0.17** ^**a/b**^	**2.52 ± 0.15** ^**a/b**^	**2.54 ± 0.01** ^**a/b**^	**2.71 ± 0.23** ^**a**^	**2.43 ± 0.07** ^**b**^	**2.14 ± 0.19** ^**c**^	**2.08 ± 0.17** ^**c/d**^	**1.83 ± 0.13** ^**d**^
3-pentanone	990	7^1^	0.25 ± 0.02	0.37 ± 0.03	0.47 ± 0.01	0.50 ± 0.04	0.24 ± 0.01	0.35 ± 0.03	0.54 ± 0.05	0.67 ± 0.04
**Ʃ C_5_/LA-Ketones**			**0.25 ± 0.02** ^**e**^	**0.37 ± 0.03** ^**d**^	**0.47 ± 0.01** ^**c**^	**0.50 ± 0.04** ^**b/c**^	**0.24 ± 0.01** ^**e**^	**0.35 ± 0.03** ^**d**^	**0.54 ± 0.05** ^**b**^	**0.67 ± 0.04** ^**a**^
Ʃ 3-ethyl-1,5-octadiene		0.014^3^	3.63 ± 0.13	3.64 ± 0.45	3.30 ± 0.12	4.44 ± 0.36	3.42 ± 0.19	2.89 ± 0.15	3.51 ± 0.49	3.84 ± 0.20
**Ʃ Penten dimers**			**3.63 ± 0.13** ^**b/c**^	**3.64 ± 0.45** ^**b/c**^	**3.30 ± 0.12** ^**c/d**^	**4.44 ± 0.36** ^**a**^	**3.42 ± 0.19** ^**b/c**^	**2.89 ± 0.15** ^**d**^	**3.51 ± 0.49** ^**b/c**^	**3.84 ± 0.20** ^**b**^
Ethyl benzene	1,140		N.D.	N.D.	N.D.	N.D.	0.55 ± 0.05	N.D.	N.D.	N.D.
Toluene	1,052		0.12 ± 0.02	0.04 ± 0.01	0.07 ± 0.01	0.12 ± 0.01	2.74 ± 0.05	0.18 ± 0.02	0.20 ± 0.03	0.08 ± 0.00
4,8-dimethyl-1,7-nonadiene	1,092		0.74 ± 0.02	0.73 ± 0.09	0.68 ± 0.00	0.90 ± 0.07	0.71 ± 0.03	0.57 ± 0.04	0.78 ± 0.13	0.82 ± 0.07
*p*-xilene	1,148		N.D.	N.D.	N.D.	N.D.	0.65 ± 0.02	N.D.	N.D.	N.D.
*m*-xilene	1,155		N.D.	N.D.	N.D.	N.D.	1.52 ± 0.08	N.D.	N.D.	N.D.
*o*-xilene	1,199		N.D.	N.D.	N.D.	N.D.	0.78 ± 0.03	N.D.	N.D.	N.D.
3-tetradecene	1,243		0.74 ± 0.03	0.73 ± 0.09	1.06 ± 0.05	1.42 ± 0.13	0.74 ± 0.06	1.20 ± 0.14	1.85 ± 0.29	1.63 ± 0.18
mesitylene	1,260		N.D.	N.D.	N.D.	N.D.	0.15 ± 0.01	N.D.	N.D.	N.D.
β-ocimene	1,265		0.30 ± 0.02	0.33 ± 0.02	0.72 ± 0.02	1.25 ± 0.12	0.41 ± 0.03	0.83 ± 0.05	1.43 ± 0.22	0.84 ± 0.07
*o*-ethyl-toluene	1,278		N.D.	N.D.	N.D.	N.D.	0.08 ± 0.01	N.D.	N.D.	N.D.
*m*-ethyl-toluene	1,298		N.D.	N.D.	N.D.	N.D.	0.35 ± 0.02	N.D.	N.D.	N.D.
Geranyl nitrile	1,319		0.39 ± 0.04	0.37 ± 0.01	0.46 ± 0.02	0.46 ± 0.05	0.44 ± 0.03	0.54 ± 0.02	0.74 ± 0.16	0.63 ± 0.08
**Ʃ Hydrocarbons**			**2.29 ± 0.09** ^**e**^	**2.20 ± 0.22** ^**e**^	**2.99 ± 0.08** ^**d**^	**4.15 ± 0.37** ^**c**^	**9.11 ± 0.35** ^**a**^	**3.32 ± 0.27** ^**d**^	**5.00 ± 0.81** ^**b**^	**4.01 ± 0.39** ^**c**^
(*E*,*E*)-2,4-hexadienal	1,466	2^1^	1.30 ± 0.09	1.26 ± 0.10	1.39 ± 0.02	1.45 ± 0.11	1.19 ± 0.01	1.14 ± 0.11	1.55 ± 0.10	1.56 ± 0.07
**Auto-oxidation**			**1.30 ± 0.09** ^**c/d**^	**1.26 ± 0.10** ^**c/d/e**^	**1.39 ± 0.02** ^**b/c**^	**1.45 ± 0.11** ^**a/b**^	**1.19 ± 0.01** ^**d/e**^	**1.14 ± 0.11** ^**e**^	**1.55 ± 0.10** ^**a**^	**1.56 ± 0.07** ^**a**^
2-methyl-butanal	922	0.0052^1^	0.08 ± 0.01	0.06 ± 0.00	0.06 ± 0.00	0.05 ± 0.01	0.07 ± 0.00	0.07 ± 0.01	0.10 ± 0.00	0.06 ± 0.00
3-methyl-butanal	927	0.0054^4^	0.04 ± 0.00	0.03 ± 0.00	0.03 ± 0.00	0.02 ± 0.00	0.04 ± 0.01	0.04 ± 0.00	0.07 ± 0.01	0.03 ± 0.00
**Amino acid metabolism**			**0.11 ± 0.01** ^**b**^	**0.09 ± 0.01** ^**c**^	**0.09 ± 0.00** ^**c**^	**0.07 ± 0.01** ^**d**^	**0.11 ± 0.01** ^**b**^	**0.11 ± 0.01** ^**b**^	**0.16 ± 0.01** ^**a**^	**0.09 ± 0.00** ^**c**^
Ethyl acetate	902	0.94^1^	0.19 ± 0.02	0.41 ± 0.02	0.38 ± 0.01	0.34 ± 0.02	0.13 ± 0.01	0.35 ± 0.04	0.41 ± 0.03	0.33 ± 0.02
Methanol	912	33^1^	0.51 ± 0.04	0.40 ± 0.02	0.29 ± 0.00	0.30 ± 0.02	0.40 ± 0.03	0.19 ± 0.02	0.38 ± 0.04	0.25 ± 0.01
Ethanol	948	30^1^	0.45 ± 0.05	0.66 ± 0.03	0.39 ± 0.01	0.51 ± 0.05	0.31 ± 0.04	0.41 ± 0.03	0.63 ± 0.05	0.47 ± 0.01
Acetic acid	1,604	0.35^5^	0.22 ± 0.03	0.20 ± 0.01	0.24 ± 0.02	0.24 ± 0.03	0.25 ± 0.01	0.20 ± 0.02	0.23 ± 0.02	0.25 ± 0.02
**Sugar fermentation**			**1.36 ± 0.13** ^**b**^	**1.67 ± 0.07** ^**a**^	**1.31 ± 0.01** ^**b/c**^	**1.40 ± 0.12** ^**b**^	**1.08 ± 0.08** ^**d**^	**1.15 ± 0.10** ^**c/d**^	**1.65 ± 0.14** ^**a**^	**1.30 ± 0.06** ^**b/c**^

OTV, olfactive threshold value. *Standard deviation; **quantified on the relative external calibration curves (paragraph 2.5), ^1^ (27); ^2^ (28); ^3^ (29); ^4^ (30); ^5^ (31). Values with the same lowercase letters in the same row have no significant differences between samples for *p* < 0.05. ND, not detected.The values in bold are the sum of the variables given above.

where z is the number of carbons of the alkane that elute before the molecule, the RT_analyte_, the RT_z_ and the RT_z + 1_ are the retention time of the analyte of interest, of the alkane that elutes before and the one that elutes after. Finally, the concentration of each volatile compounds was determined according to the formula:


$$C_{c}=\left(A_{c}/A_{\mathrm{IS}}\right)/m_{a}\text{,}$$

where C_c_ is the concentration of the compound of interest; A_c_ is the area of the compound of interest; A_IS_ is the area of the IS; m_a_ is the slope of the related external standard calibration curve of each representative compound. The calibration curves were built in the range 0.05–25 mg/kg for each volatile compound (see Section 2.1) as these molecules are representative of each main chemical class of compounds generally present in VOOs.

### 2.6 Phenolic compound analysis

#### 2.6.1 Phenolic extraction

Polar phenolics were extracted from two grams of each EVOO samples with a hydroalcoholic mixture and according to the procedure reported by the IOC (32). A syringic acid solution (c = 0.015 mg mL^−1^) was used as internal standard. About 1.5–2 mL of the phenolic fraction were stored in a PP centrifuge microtube at −18°C before HPLC analyses. The same procedure was repeated on two grams of EVOO except replacing 1 mL of standard solution with 1 mL of methanol/water 4/1 (v/v). On this second extract, the determination of the total phenolic content by UV/VIS spectrophotometry and acid hydrolysis of phenolics was performed. Each extraction procedure was carried out three times on different EVOO samples.

#### 2.6.2 Colorimetric determination of the total phenolic content (TPC) by UV/VIS spectrophotometry

Colorimetric determination of the total phenolic content (TPC) was measured following the Folin-Ciocalteau procedure as reported by Singleton and Rossi (33) and described as follows. 7.3 mL of water, 0.2 mL of EVOO hydroalcoholic extract, 0.5 mL of Folin-Ciocalteau reagent, and 2.0 mL of 15% (w/v) sodium carbonate in water were transferred to a 10-mL PTFE screw cap glass tube and shaken by hand for 5 s. The mixture was then kept in the dark at room temperature. After at least 2 h but not more than 8 h, the absorbance of the solution was read at 750 nm in a single beam spectrophotometer (mod. UV-5600) from Hinotek (Ningbo, China). TPC was calculated by a gallic acid calibration curve. From a stock solution (c = 2.03 mg mL^−1^) in methanol/water 4/1 (v/v), diluted solutions were prepared in the same solvent mixture in a concentration range of 0.0025–0.25 mg mL^−1^ (seven calibration points, *r*^2^ > 0.99). Observed absorbance values were corrected by subtracting the absorbance of a blank sample replacing the hydroalcoholic extract with 0.2 mL of methanol/water 4/1 (v/v). Extracts, gallic acid standard solutions, and blanks were analyzed in two replications.

#### 2.6.3 Determination of phenolic profile by high performance liquid chromatography (HPLC)

One mL of each extract added with internal standard was filtered in a HPLC glass vial through a 3-mL plastic syringe by a PVDF filter (diameter: 13 mm, pore dimension: 0.45 μm). Phenolic extracts were analyzed in a linear elution gradient mode on a Nexera™ Series ultra-high performance liquid chromatograph from Shimadzu (Kyoto, Japan) equipped with a solvent delivery module including two binary pumps and a degassing unit (mod. LC-40Bx3), a system controller (mod. CBM-40), a UV–VIS photodiode array detector (mod. SPD-M40), an autosampler for liquid samples (mod. SIL-40Cx3), and a column oven (mod. CTO-40S). Mobile phases were: (A) 0.2% (v/v) orthophosphoric acid in water, and B) acetonitrile/methanol 1/1 (v/v). Methanol/water 4/1 (v/v) was used as cleaning solution for the autosampler syringe before and after sample injection. The chromatographic separation was carried out at 35°C by a SphereClone™ 5 μm ODS(2) 80 Å LC column (250 × 4.6 mm i.d.), fitted with a guard cartridge Gemini C18 (4 × 3.0 mm i.d.), both from Phenomenex (Torrance, CA, USA). Other chromatographic conditions (gradient program, flow rate, injection volume) were the same described by IOC (26). HPLC traces were acquired at 280 nm, whereas absorption spectra were recorded from 190 to 400 nm. Data were stored and processed by the software LabSolutions (ver. 5.97) from Shimadzu. The calculation of response factors of the external calibration standards (RFs) and the ratio syringic acid-to-tyrosol response factors (RRF) were performed as suggested by IOC (26). RRF was equal to 4.9 and lay within the range proposed by the IOC (32). The relative retention time (RRT) of each peak was calculated with respect to the retention time of syringic acid and compound identification was carried out comparing RRTs and UV spectra with data reported by the IOC (32). The content of each phenolic compound herein identified was calculated considering the compound peak area, the amount, and the peak area of syringic acid corrected by RRF (32). Each extract and standard solution were injected twice. Standard solutions were filtered before HPLC analyses on PVDF filters as EVOO extracts.

#### 2.6.4 HPLC determination of hydroxytyrosol (HYTY) and tyrosol (TY) after acid hydrolysis

The total amount of HYTY and TY in EVOO samples was determined according to a “fit for purpose” analytical procedure described by Tsimidou et al. (34), with some modifications, and based on three successive steps: (a) acid hydrolysis carried on hydroalcoholic extracts with some modifications; (b) successive HPLC analysis; (c) data analysis that considered free and bound form of HYTY and TY and applied proper correction factors. A volume (0.4 mL) of each polar extract was mixed with 0.4 mL of 1 M H_2_SO_4_, briefly shaken on a vortex stirrer, and incubated in a water bath at 80°C for 2. The hydrolysate extract was cooled at room temperature, diluted with 0.4 mL of methanol/water 4/1 (v/v), and then filtered in a HPLC glass vial through a 3-mL plastic syringe by a PVDF filter (diameter: 13 mm, pore dimension: 0.45 μm) before injection. The same liquid chromatograph, mobile phases and cleaning solution used in the determination of individual phenolic profile and formerly described were employed. The chromatographic separation was carried out under controlled temperature at 35°C by a Shim pack XR-ODS III column 1.6 μm (75 × 2.0 mm i.d.) from Shimadzu, fitted with a guard cartridge Gemini C18 (4.0 × 3 mm i.d.) from Phenomenex. The gradient elution was as follows: 0–7 min, 96 to 65% A; 7–13.5 min, 65 to 0% A; 13.5–19 min, 0% A; 19–20 min 0 to 96% A; 20–40 min, 96% A. The flow rate was 0.3 mL min^−1^ and the injection volume was 5 μL. HPLC traces were acquired at 280 nm, whereas absorption spectra were recorded from 190 to 400 nm. Data were processed with the software LabSolutions (ver. 5.97) from Shimadzu. HYTY and TY were quantified by external standard mode constructing two calibration curves, one for each analyte. Two stock solutions were prepared in methanol/water 4/1 (v/v) at a concentration of 0.50 and 1.52 mg mL^−1^ for HYTY, and TY, respectively. Diluted standard solutions containing both compounds were then prepared by serial dilution in a concentration range from 0.001 to 0.050 mg mL^−1^ (six calibration points, *r*^2^ > 0.99). Each extract and standard solution were injected twice. The total amount of HYTY and TY was calculated as the sum of their free and bound forms. The amounts of free HYTY and TY were previously assessed in the determination of individual phenolic profile by HPLC (internal standard mode). To determine the actual amount of bound HYTY and TY, the content of free HYTY and TY was subtracted by the HYTY and TY content of hydrolysate extracts determined by calibration curves. Two corrections factors, 2.2 and 2.5 for HYTY and TY, respectively, were applied to the bound amounts of the two compounds. These factors were determined by Tsimidou et al. (34) dividing the mean molecular mass (343 amu) of the most known bound forms of HYTY and TY by the molecular mass of HYTY (154 amu) and ΤYT (138 amu), respectively. The following formulas explains the calculation above described.


$$\begin{array}{l} \mathrm{Total}\;\mathrm{HYTY}\;\mathrm{and}\;TY={\mathrm{HYTY}}_{\mathrm{free}}+{TY}_{\mathrm{free}}\\ \;+{\mathrm{HYTY}}_{\mathrm{bound}}+{TY}_{\mathrm{bound}}\text{,} \end{array}$$

where:


$$\begin{array}{l} {\mathrm{HYTY}}_{\mathrm{bound}}=2.2\times \left({\mathrm{HYTY}}_{\mathrm{hydrolysate}}-{\mathrm{HYTY}}_{\mathrm{free}}\right)\;\mathrm{and}\;\\ \;{TY}_{\mathrm{bound}}=2.5\times \left({TY}_{\mathrm{hydrolysate}}-{TY}_{\mathrm{free}}\right)\text{.} \end{array}$$

### 2.7 Statistical analysis

Data analysis was carried out with Microsoft® spreadsheet program 2016 (Microsoft Corp., Redmond, WA). Analysis of variance (*p* < 0.05) was carried out with XLSTAT (Addinsoft Corp., Paris, France).

## 3 Results and discussion

### 3.1 Jaèn maturity index

**Figure 1:**
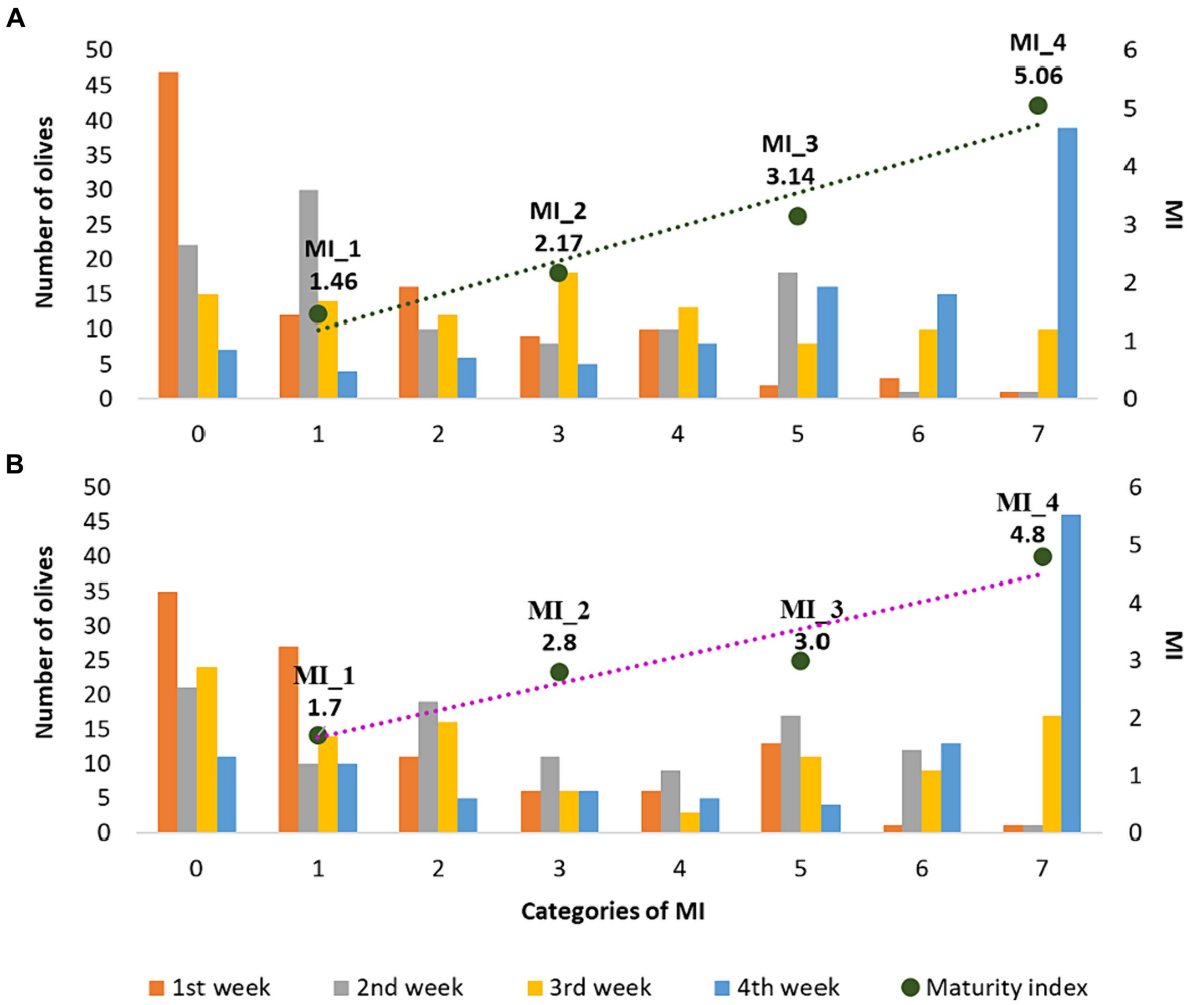
Number of olives deriving from integrated pest management **(A)** and organic farming **(B)** in the seven classes of the Jaèn MI (histograms) and evolution over the four weeks of harvesting.

To monitor the degree of ripeness of the olive fruits, at the beginning of each of the four week periods, the Jaèn MI test was performed on the olives obtained through integrated pest management (A) and organic farming (B). During the ripening process, xanthopylls accumulate in the drupes while the oil content increases. As ripening progresses, photosynthetic activity decreases and the concentrations of both chlorophylls and carotenoids progressively decrease (35). Toward the end of ripening, the fruit becomes violet or purple due to the accumulation of xanthopylls (36). Proper sampling before the traditional drupe maturation period can obtain olive oils that best express their sensory and health-promoting characteristics, thus obtaining the greatest possible quantity of oil with the highest added value (19). Moreover, the compositional diversity of EVOOs reflects several variables. Among these, the MI of the olive fruits is considered an extremely relevant factor and it is essential to know and control its effect on the final product (4). In fact, from a previous study it emerged that the ripeness stage of *Nostrana di Brisighella* olives from which the best oil is obtained corresponds to a Jaèn index value between 2.5 and 3.5. EVOOs produced from olives harvested within this time frame have an improved sensory profile accompanied by excellent chemical and nutritional properties (37). In this research, since the COI/OH/Doc. No 1 (19) does not provide for replicates, the results are reported on a representative sample of 100 olives that were taken from several trees with a comparable crop load of homogeneous maturity (color). Regarding the weekly collections, the olives were harvested by the same person in a very similar way, taking them from the same trees in the same quantity and with the same methods according to the protocol. In light of this, the temporal progression of the harvesting season was accompanied by a linear increase in the MI for olives coming from both agronomic systems used as shown in Figure 1. In fact, an increase in the Jaèn index was highlighted in the four week periods for olives from integrated pest management (from 1.46 to 5.06) and for olives from organic farming (from 1.69 to 4.78). A similar trend was also shown in the study by Dag et al. (38), where the influence of harvest date on the MI of two different cultivars (*Barnea* and *Souri*) was evaluated in three different olive oil campaigns (2005/2006, 2006/2007, 2008/2009).

### 3.2 Sensory analysis

From sensory results, all the samples analyzed belonged to the commercial category “extra virgin olive oil” as no sensory defects were detected. Several authors have reported that the ripening of olives, as well as the agronomic techniques adopted, have a marked impact on the sensory attributes of olive oil (39, 40) although in the study conducted by Morrone et al. (41) none of the sensory descriptors were affected by MI. The results obtained in the present analysis and shown in Table 2 demonstrate that the variables considered had an impact on the sensory characteristics of the EVOOs analyzed. In particular, sample “1B” differed from other samples due to the presence of the secondary sensory attribute resembling tomato.

**Table 2:** Results of the sensory analysis (Panel test) carried out by the DISTAL Professional Committee according to the list of descriptors established for PDO EVOOs (23).

Sensory attributes	Sample							
	1A	2A	3A	4A	1B	2B	3B	4B
Fruity	5.3	4.5	4.9	5.1	5.5	5.0	3.8	4.5
Bitter	4.8	5.0	4.5	5.0	5.7	5.5	4.0	3.9
Pungent	5.5	5.0	4.2	4.7	5.0	5.2	4.0	4.0
Grass	4.0	2.9	2.0	3.0	3.5	3.0	0	1.8
Artichoke	3.0	3.0	3.0	3.1	3.5	3.0	0	2.0
Tomato	0	0	0	0	3.5	0	0	0

**Figure 2:**
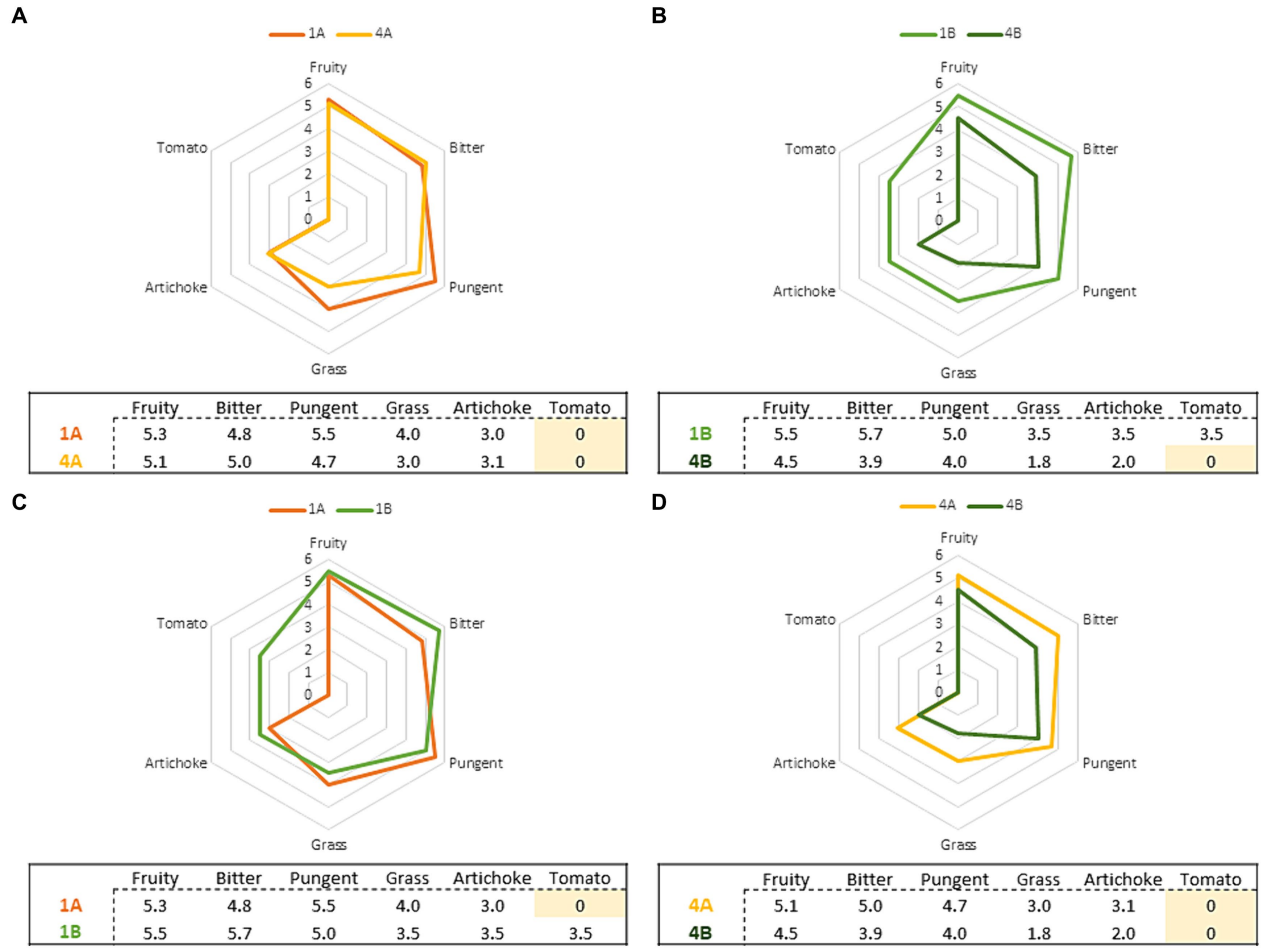
Comparison of the sensory profiles relating to the EVOOs according to the harvest period [**(A,B)** 1A and 1B are EVOOs produced from olives harvested in the first week; 4A and 4B are EVOOs produced from olives harvested in the fourth week] and of the agronomic system [**(C,D)** 1A and 4A are EVOOs produced from olives obtained from integrated pest management, 1B and 4B are EVOOs produced from olives obtained from organic farming].

A study conducted on the same *Nostrana di Brisighella* cultivar highlighted the influence of the harvest period, and therefore on the MI of the drupes, on the sensory profiles of the olive oils obtained (37). Specifically, a decline in the intensity of the positive attributes was observed as the MI of the drupes increased (even though the median of the intensity remained in the medium category, i.e., between 3 and 6), and in particular with a MI higher than 3.5 which, in this case, was obtained in the fourth week of harvesting for both agronomic systems adopted. Similarly to the aforementioned work, it was also observed herein that “fruity,” “bitter,” and “pungent” tended to have a higher intensity in the EVOOs produced in the first week of harvest, and that these attributes decreased variably in samples obtained from riper olives. To better show these differences, pairwise comparisons were made between the following samples: 1A *vs* 4A, 1B *vs* 4B, 1A *vs* 1B, 4A *vs* 4B. These comparisons made it possible to underline the effect of the two distinct agronomic systems used (integrated pest management and organic farming) and of the harvesting period on the differences in the sensory profile of the olive oils produced. The results, shown as radar charts (Figure 2), relate the positive sensory attributes of “fruity,” “bitter,” “pungent,” “grass,” “artichoke” and, for sample 1B, also the secondary sensory note of “tomato.” Figures 2A,B refer to the influence of the harvest period on the sensory characteristics of the EVOOs produced, while Figures 2C,D investigate the effect of the agronomic system used (integrated pest management and organic farming).

From the sensory data, it was possible to observe that as the drupe harvesting time increased, and consequently their MI, there was a decrease in the intensity of the sensory attributes perceived for the EVOOs obtained from olives grown through both integrated pest management and organic farming. These results agree with what emerged from previous research carried out on *Nostrana di Brisighella* oils produced with olives with an increasing MI (37) as well as virgin olive oils produced from the *Oblica* cultivar, where as the olives ripened, the intensity of the “fruity” attribute decreased. However, the same attribute showed a different trend as a function of MI for the *Leccino* cultivar, following a Gauss curve (4). Volatile molecules, particularly C_5_ and C_6_ compounds, are responsible for the positive sensory attributes of EVOOs and are released during the extraction of olives in the lipoxygenase (LOX) pathway (4). The concentration of the latter decreases with maturation (42), following a reduction in enzyme activity with ripening, and are highly correlated with the “fruity” attribute (43, 44). The formation of these molecules is associated with the content of polyunsaturated fatty acid (PUFA), since they are a substrate of lipoxygenase, while phenolic compounds inhibit the same enzymes (45). Furthermore, climatic and agronomic conditions of olive growing can affect the volatile composition and consequently the sensory profile of olive oils obtained from the same cultivar (46, 47). In fact, in Figure 2A a different trend was observed for organic EVOO (B) compared to that obtained from integrated pest management (A): for the former, a greater decline in the intensity of the perceived sensory attributes was detected from the first to the fourth week of harvesting (2.1 average points for B and 0.7 for A). In contrast, the type of agronomic system adopted does not seem to influence the sensory profile of the EVOOs produced. Comparing the first and fourth weeks (Figure 2B), the organic EVOO produced in the first week was characterized by more intense sensory attributes compared to the fourth where more feeble sensory attributes were highlighted. Finally, EVOOs produced from integrated pest management showed a more constant trend in the intensity of sensory attributes over the four week periods compared to the organic ones (Figure 2D), which were characterized by greater variability. These trends can be partly explained by the different phenolic content of the olive oils obtained from the two agronomic systems; this can be attributable to the balance between adequate nutrition of sustainable crops and the pathogenic pressure that led to the synthesis of phenolic compounds (48). Indeed, a higher polyphenolic content in sustainable *vs* organically produced crops was reported by Asami et al. (48) and by Chinnici et al. (49) in the case of *Golden Delicious* apples produced by both organic and integrated agricultural methods.

### 3.3 Volatile compound analysis

**Figure 3:**
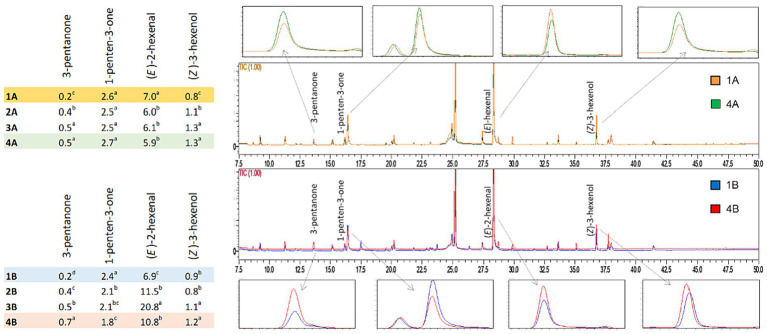
Comparison of chromatograms obtained from volatile compound analysis of EVOO samples derived from the two agronomic systems (integrated pest management: 1A *vs* 4A; organic: 1B *vs* 4B).

The EVOO samples were obtained from olives in excellent condition and processed on the same day of harvest. Indeed, to obtain high quality extra virgin olive oil (premium oil), olives must be processed as quickly as possible after harvesting. Normally, olive fruits should be delivered to the mill within a day of harvesting to limit oxidation and degradative phenomena (50). This, together with proper processing of the olives in the mill, provides an EVOO aromatic fraction composed mainly of C_5_ and C_6_ compounds derived from polyunsaturated fatty acids through the LOX pathway. C_6_ and C_5_ compounds are responsible for the peculiar profile of high-quality EVOOs that is highly appreciated by consumers for their sensory, nutritional, and health-protecting properties (51). The ripening stage of olives is also a crucial parameter in the formation of volatile compounds via the LOX pathway, with an increase in the activity of peroxidase and β-glucosidase during olive ripening (52). During the initial phase of inolition, olives contain practically equal quantities of C_6_ aldehydes and C_6_ alcohols and almost all C_6_ aldehydes reach their maximum concentration in the subsequent veraison stage (53). Twenty-five volatile compounds were identified and quantified in samples. The only exception was recorded in the case of organic oil produced from drupes harvested in the first week (1B), for which 7 additional volatile compounds were detected. The obtained quali-quantitative results are very similar; in Figure 3 selected volatile compounds originating from the LOX pathway are highlighted. Specifically, C_5_ compounds (3-pentanone, 1-penten-3-one) responsible for the sensory attribute of fruity and green leaf, and C_6_ compounds ((*E*)-2-hexanal, (*Z*)-3-hexenol) responsible for green notes (54).

The table in Figure 3 highlights differences in the concentration of some volatile compounds. In particular, (*E*)-2-hexenal is present in highest concentrations among the other volatile compounds for all samples analyzed, and responsible for the positive “green fruity” attribute (44). The highest concentration (20.80 mg/kg) was found in the sample obtained in the third week from organic farming (3B). (*Z*)-3-hexenol reached higher concentrations in the oils obtained in the last few weeks of harvest. This molecule is associated with “green,” “fruity,” “bitter,” and “aromatic herb” notes (55). Among C_5_ ketones, 3-pentanone and 1-penten-3-one showed significant differences between olive harvest weeks, with the exception of 1-penten-3-one deriving from integrated pest management which did not change significantly. According to the literature, these compounds are related to aromatic notes of “bitter,” “fruity” and “pungent” (27, 56–58). As reported above, regarding the organic oil of the first week (1B), ethyl-benzene, *p*-xylene, *m*-xylene, *o*-xylene, mesitylene, *o*-ethyl-toluene, and *m*-ethyl-toluene were also identified and quantified in a range between 0.08 to 1.52 mg/kg, which were not found in any other sample. These molecules are part of a group of volatile compounds called BTEXS, which are toxic pollutants released into the environment. Their presence can be due to both natural (e.g., superior plants wax, natural oil seepage) or anthropogenic factors (e.g., combustion products of wood and fuels) and the lipophilic structure makes them especially harmful by accumulation in matrices containing fat. As highlighted by a literature survey on virgin olive oils from the European Union, the concentration ranges of BTEXS can be wide depending on numerous variables, also in relation to the different analytical conditions applied (59).

**Figure 4:**
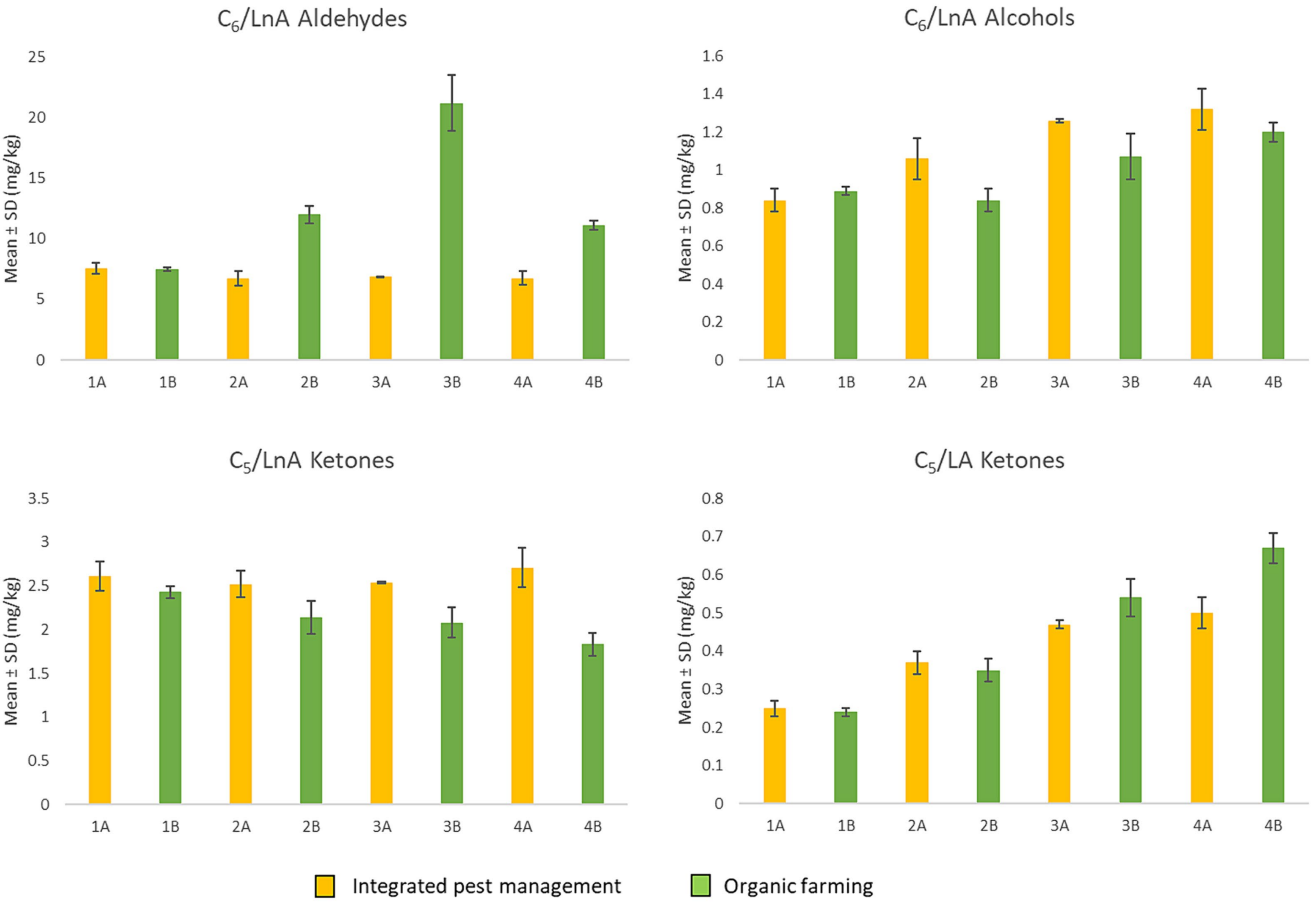
Sum of C_6_ aldehydes and alcohols from LnA and C_5_ ketones from LA and LnA through the LOX pathway according to the harvest period (from the first to the fourth week of harvest) and of the agronomic system (integrated pest management and organic farming).

Despite the diverse concentrations at which the different volatile compounds may be present, each molecule is characterized by a specific odor threshold value (OTV), which is defined as the lowest concentration recognizable by the human sense of smell (44). Table 1 shows the OTV of the C_5_ and C_6_ compounds, formed during the LOX pathway starting from linoleic (LA) and linolenic (LnA) acids, and the minor volatile compounds detected in all samples, reported as sum of the chemical classes to which they belong (53). For this reason, Figure 4 shows the significantly more relevant trends of the summations of some C_5_ and C_6_ volatile compounds, which are responsible for positive sensory attributes.

As can be seen in Figure 4, for the C_6_ aldehydes derived from LnA, no significant differences were found in the first week of harvest for either agronomic system. Subsequently, from the second week onwards, regarding the organic EVOOs, an increase in these compounds was noted, highlighting higher levels compared to the oil obtained from integrated pest management (A). C_6_ alcohols do not show the same trend highlighted by the aldehydes, although there are no significant differences for the first week of harvest. For the organic EVOOs (B) produced in the second and third weeks, this class of compounds shows lower concentrations, with no significant differences in the fourth week. In general, the increase in these compounds appears to be associated with the action of enzymes that catalyze the reduction of aldehydes (44).

For C_5_ compounds, ketones originating from LnA (1-penten-3-one) did not show significant differences for the first week of harvest. From the second week onwards, those derived from oil obtained by integrated pest management (A) tended to remain constant while for organic EVOOs (B) a progressive decrease was observed. The decrease in 1-penten-3-one during olive ripening was also highlighted in the study by Lukić et al. (60), in which the authors consequently also found a reduction in “green” notes. This trend is in line with that observed herein, which could explain the reduction in the “bitter,” “pungent,” and “fruity” attributes perceived during sensory analysis.

### 3.4 Total phenolic content (TPC)

The agronomic system, olive ripening stage, and their interaction significantly affected TPC as shown in Table 3. On average, the TPC was 14% higher in integrated pest management derived EVOOs compared to organic ones and significant differences were also noticed between the two types of oils within each ripening stage. Important differences were assessed at the third and fourth harvest week, where 3A and 4A oils were 27 and 23% higher, respectively, in TPC than the corresponding organic 3B and 4B samples. A progressive and consistent decrease occurred from the first to the fourth harvest week, where the mean TPC was lower than 300 mg of gallic kg^−1^ of oil. A more significant ripening effect was verified in organic samples than in those obtained from integrated pest management, in particular in EVOOs derived from olive fruits collected at the third and fourth harvest week. TPC decreased by 1% (not statistically significant), 11, and 24% in samples 2A, 3A, and 4A, respectively, compared to sample 1A, where a 6, 27, and 35% reduction in TPC was seen in organic samples 2B, 3B, and 4B, respectively, compared with sample 1B. The data from the present study agree with those reported by Rotondi et al. (37) in an investigation evaluating the effect of ripening on the quality of *Nostrana di Brisighella* EVOO. In that research, total phenolics more than halved from the first to the fourth stage and were present at levels of 441, 380, 277, and 210 mg gallic acid kg^−1^ of oil at the four consecutive MIs. With regard to the effect of growing system on TPC, controversial results were reported by Ninfali et al. (61) in a 3-year study on the quality of Italian EVOOs from olive fruits of cv. *Leccino* and *Frantoio*. In organic *Leccino* EVOOs, phenols were higher than in conventional samples only in the second year (+22%), whereas in the third year, a higher phenolic content was observed in conventional EVOO (+12) while no significant difference were seen in the first year. Regarding *Frantoio* EVOOs, organic samples were higher in phenolics in comparison to conventional samples in the first year (+35), whereas in the second year a higher amount was present in conventional oil (+41%), and no difference was observed in the third year. The UV/VIS spectrophotometric assay used herein allows a non-specific quantification of phenolic compounds (62, 63) that can also include other possible reducing non-phenolic substances (also if in EVOOs ascorbic acid, amines, or sugars are not present). Nevertheless, the Folin-Ciocalteau procedure is a widespread analytical protocol in the overall evaluation of the total amount of phenolic compounds in olive oils, especially for its ease of use.

**Table 3:** Total amount of phenolics and secoiridoids of EVOO samples obtained by different analytical procedures employed in the present study.

Samples	TPC^a^	PHEN-TOT^b^	SEC-TOT^c^	PHEN-TOT-HYDR^d^	PHEN-TOT-HYDR^d^
	mg gallic acid kg^−1^ oil	mg kg^−1^ oil	mg 20 g^−1^ oil	mg kg^−1^ oil	mg 20 g^−1^ oil
Agronomic system					
Integrated pest management (A)	387.9	300.8	5.0	473.7	9.5
Organic (B)	338.9	277.4	4.5	440.3	8.8
Significance^e^	**	**	**	**	**
LSD^f^	4.8	2.9	<0.1	13.6	0.3
Ripening stage (harvest week)					
1	417.0	326.0	5.3	531.9	10.6
2	401.5	321.3	5.4	511.7	10.2
3	340.1	279.5	4.6	426.2	8.5
4	295.1	229.6	3.7	358.2	7.2
Significance	**	**	**	**	**
LSD	6.8	4.1	0.1	19.3	0.4
Agronomic system × ripening stage					
1A	426.0	334.4	5.6	526.0	10.5
2A	419.9	327.6	5.5	535.9	10.7
3A	380.6	295.1	4.9	454.8	9.1
4A	325.3	246.1	4.0	378.0	7.6
1B	407.9	317.7	5.1	537.7	10.8
2B	383.2	315.0	5.2	487.5	9.7
3B	299.7	263.8	4.3	397.5	8.0
4B	264.9	213.1	3.5	338.3	6.8
Significance	**	**	**	**	**
LSD	9.6	5.8	0.1	27.3	0.5

^a^TPC: total phenolic content determined by colorimetric Folin-Ciocalteau method. ^b^Total phenolic content determined by HPLC on polar extracts recovered from EVOO samples (no acid hydrolysis). ^c^Total amount of secoiridoid compounds determined by HPLC (no acid hydrolysis). ^d^Sum of the amount of hydroxytyrosol (HYTY) and tyrosol (TY) determined by HPLC after acid hydrolysis on polar extracts. ^e^*p ≤ 0.05; **p ≤ 0.01; NS, not significant. ^f^LSD, least significant difference (*p* = 0.05).

### 3.5 Determination of polar phenolics by HPLC

**Figure 5:**
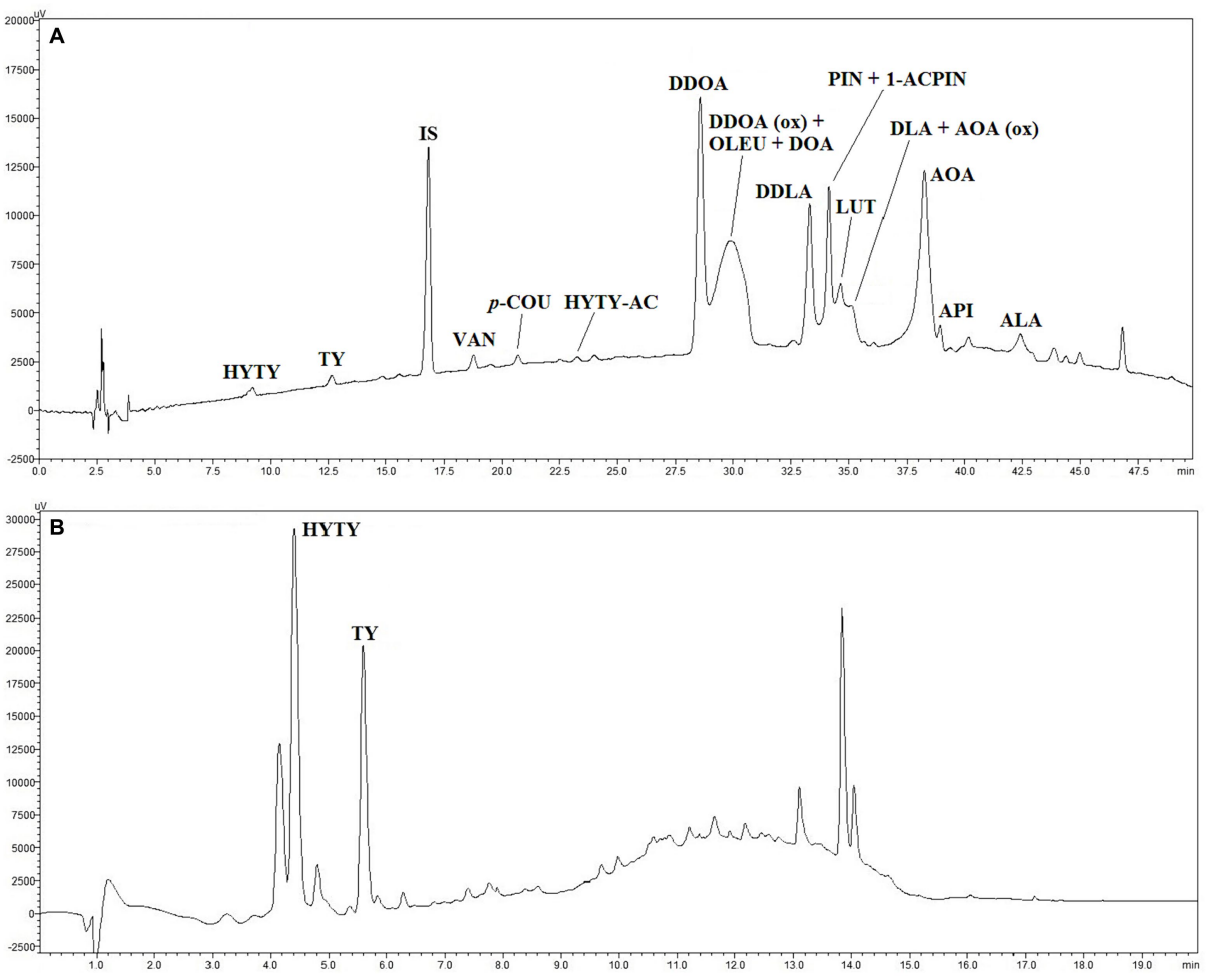
HPLC traces of polar phenolic extracts obtained from EVOO sample 1A without **(A)** and after acid hydrolysis **(B)**. For individual compound names see Table 4, for experimental conditions see “Materials and methods” paragraph. IS (trace A), internal standard (syringic acid).

Compounds identified in HPLC traces of hydroalcoholic extracts recovered from EVOOs and not subject to acid hydrolysis are reported in Table 4 with the corresponding working tags and retention times. A typical HPLC trace is shown in Figure 5: despite effective separation for most compounds, some, in particular the secoiridoids (complex forms containing hydroxytyrosol and tyrosol), were still co-eluting and not clearly separated from each other. Trends formerly discussed for TPC data were confirmed by HPLC analyses. In fact, the total amount of phenolics determined by HPLC was highly related to TPC data (*r*^2^ = 0.879, *p* < 0.001). Integrated pest management led to a higher amount of phenolics (+8% on average) in comparison to organic farming and a significant decrease in phenolics was noticed from the first to the last ripening stage (Table 3). A greater decrease during ripening in organic *vs* integrated pest management samples was also assessed by HPLC: in comparison to sample 1A, the sum of individual phenolics was lower in samples 3A and 4A (−12% and − 26% lower, respectively), whereas samples 3B and 4B showed a phenolic amount that was −17% and − 33% lower than sample 1B. The amount of phenolics determined by HPLC was in the range reported by Barbieri et al. (17) where the same analytical procedure was performed on 10 samples of *Nostrana di Brisighella* EVOOs. In fact, in that study, polar phenolics ranged from 256 to 434 mg kg^−1^ of oil. Most of the compounds identified in the present research were secoiridoids, representing on average more than 80% of the total phenolics in integrated pest management and organic samples, whereas lignans, phenolic acids, and flavonoids were present in lower relative amounts (Table 5). The highest relative contents were seen for compounds DDOA, AOA and co-eluting DDOA (ox), OLEU, and DOA, all accounting for more than 15% of phenols (Table 5). Hydroxytyrosol (HYTY) and tyrosol (TY) were not detected in the first stage of ripening and were present in very low amounts only after the third week of harvest; luteolin was the predominant flavonoid in both types of samples, amounting to more than 70% of this class of phenolics (Table 5). Some small but significant differences were noticed between integrated pest management and organic samples and among ripening stages, within the same type of oil, and EVOOs obtained from the two types of growing systems showed a similar phenolic profile. A recent comparison between two kind of EVOOs was carried out by López-Yerena et al. (64). In that investigation, a higher amount of total phenolics (+35%) were detected by HPLC (no hydrolysis on polar extracts) in organic than in conventional samples from Spain, with phenols at levels of 457 and 338 mg kg^−1^ of oil, respectively.

**Table 4:** Names, working tags, chemical classes, retention times, and amount ranges of individual phenolic compounds identified and quantified by HPLC in polar extracts recovered from EVOO samples.

Compound name	Tag	Chemical class	Retention time (min)^a^	Amount ranges (mg kg^−1^ oil)^c^
Hydroxytyrosol	HYTY	Simple phenols	9.20^a^	TR-0.6
Tyrosol	TY	Simple phenols	12.67^a^	TR-0.4
Vanillin	VAN	Simple phenols	18.77	TR-1.6
*p*-Coumaric acid	*p*-COU	Phenolic acids	20.69	ND-TR
Hydroxytyrosyl acetate	HYTY-AC	Simple phenols	23.20	ND-TR
Decarboxymethyl oleuropein aglycone, dialdehyde form	DDOA	Secoiridoids	28.57	38.3–67.9
Decarboxymethyl oleuropein aglycone, oxidized dialdehyde form; oleuropein; oleuropein aglycone, dialdehyde form	DDOA (ox) + OLEU + DOA^b^	Secoiridoids	29.86	51.9–100.8
Decarboxymethyl ligstroside aglycone, dialdehyde form	DDLA	Secoiridoids	33.31	21.9–42.4
Pinoresinol; 1-acetoxy-pinoresinol	PIN +1-ACPIN^b^	Lignans	34.15	24.7–34.6
Luteolin	LUT	Flavonoids	34.65	11.1–25.0
Ligstroside aglycone, dialdehyde form; oleuropein aglycone, oxidized aldehyde and hydroxylic form	DLA + AOA (ox)^b^	Secoiridoids	35.14	6.0–10.2
Oleuropein aglycone, aldehyde, and hydroxylic form	AOA	Secoiridoids	38.26	42.3–75.7
Apigenin	API	Flavonoids	38.93	2.9–3.9
Ligstroside aglycone, aldehyde, and hydroxylic form	ALA	Secoiridoids	42.42	TR-8.9

^a^Retention times determined on traces derived from HPLC analyses of extracts that did not undergo acid hydrolysis. In HPLC traces of hydrolysate extracts, the retention times of HYTY and TY were 4.41 and 5.60 min, respectively. ^b^Co-eluting compounds. ^c^ND, not detected; TR, traces.

**Table 5:** Relative amount of individual phenolics, secoiridoids, and non-secoiridoid phenolics determined by HPLC (no acid hydrolysis performed on polar extracts).

Samples	Relative amount (mg mg^−1^ of phenolics)^a^															
	HYTY	TY	VAN	*p*-COU	HYTY-AC	DDOA	DDOA (ox) + OLEU + DOA	DDLA	PIN + 1-ACPIN	LUT	DLA + AOA (ox)	AOA	API	ALA	SEC-TOT	OTHER
Agronomic system																
Integrated pest management (A)	0.001	TR	TR	TR	ND	0.167	0.309	0.089	0.103	0.058	0.030	0.219	0.013	0.012	0.826	0.174
Organic (B)	TR	0.001	0.001	TR	TR	0.193	0.256	0.136	0.109	0.060	0.028	0.186	0.013	0.018	0.817	0.183
Significance^b^	NS	*	**	NS	NS	**	**	**	**	**	**	**	NS	**	**	**
LSD^c^	-	0.001	0.001	-	-	0.001	0.002	0.001	0.001	0.001	0.001	0.002	-	0.002	0.001	0.001
Ripening stage (harvest week)																
1	TR	TR	0.002	TR	ND	0.170	0.272	0.113	0.101	0.069	0.031	0.206	0.010	0.025	0.817	0.183
2	TR	TR	TR	ND	ND	0.167	0.310	0.102	0.098	0.057	0.029	0.206	0.012	0.018	0.833	0.167
3	TR	0.001	TR	TR	TR	0.213	0.266	0.121	0.105	0.056	0.026	0.189	0.014	0.009	0.825	0.175
4	0.001	0.001	TR	TR	ND	0.169	0.280	0.114	0.119	0.054	0.029	0.210	0.016	0.007	0.811	0.189
Significance	NS	NS	**	NS	NS	**	**	**	**	**	**	**	**	**	**	**
LSD	-	-	0.001	-	-	0.002	0.002	0.001	0.001	0.001	0.001	0.002	0.000	0.003	0.001	0.001
Agronomic system × ripening stage																
1A	TR	TR	TR	TR	ND	0.181	0.293	0.099	0.093	0.060	0.031	0.211	0.011	0.021	0.836	0.164
2A	TR	TR	TR	ND	ND	0.163	0.300	0.088	0.095	0.061	0.030	0.231	0.012	0.020	0.832	0.168
3A	TR	TR	TR	TR	ND	0.169	0.326	0.081	0.102	0.056	0.029	0.218	0.013	0.006	0.829	0.171
4A	0.002	TR	TR	TR	ND	0.156	0.315	0.089	0.121	0.056	0.029	0.216	0.016	TR	0.808	0.192
1B	TR	TR	0.005	TR	ND	0.159	0.251	0.127	0.109	0.079	0.032	0.201	0.009	0.028	0.798	0.202
2B	TR	TR	TR	ND	ND	0.172	0.320	0.116	0.101	0.054	0.029	0.180	0.012	0.016	0.833	0.167
3B	TR	0.001	TR	TR	TR	0.257	0.207	0.161	0.108	0.055	0.023	0.160	0.015	0.011	0.821	0.179
4B	TR	0.002	TR	ND	ND	0.182	0.244	0.140	0.116	0.052	0.029	0.204	0.016	0.015	0.815	0.185
Significance	NS	NS	**	NS	NS	**	**	**	**	**	**	**	**	**	**	**
LSD	-	-	0.001	-	-	0.002	0.003	0.002	0.001	0.001	0.001	0.003	0.001	0.004	0.002	0.002

^a^For individual compound names see Table 3; SEC-TOT, total secoiridoids (sum of HYTY, TY, HYTY-AC, DDOA; DDOA (ox) + OLEU + DOA, DDLA, PIN + 1-ACPIN, DLA + AOA (ox), AOA, ALA; OTHER, non-secoiridoid phenolic compounds; TR, traces; ND, not detected. ^b^*p ≤ 0.05; **p ≤ 0.01; NS, not significant. ^c^LSD, least significant difference (*p* = 0.05).

### 3.6 Determination of polar phenolics by HPLC after acid hydrolysis on hydroalcoholic extracts

This HPLC method, based on acid hydrolysis on hydroalcoholic extracts and proposed by Tsimidou et al. (34), was applied with some modifications. While not allowing a complete profiling of all individual compounds compared to the IOC (32) method, this procedure enables accurate quantification of HYTY and TY in both free and bound forms by the application of proper correction factors, which is useful to make the polyphenol health claim (EU Reg, 432/2012). An HPLC trace of a hydrolysate extract is shown in Figure 5. Phenolic content after acid hydrolysis was highly related to TPC (*r^2^* = 0.807, *p* < 0.001) and the total phenolic amount assessed on extracts by HPLC without hydrolysis (*r^2^* = 0.744, *p* < 0.001). The samples from the integrated pest management system on average were higher in phenolics than organic samples (+8%) and organic EVOOs were subject to a more consistent decrease in phenolics than other samples during ripening (Table 3). In reality, the sum of total HYTY and TY in sample 2A was not significantly different in comparison to 1A, whereas samples 3A and 4A were 14 and 28% lower in phenolics than sample 1A. In organic EVOOs, more substantial modifications took place: samples 2B, 3B, and 4B were 9, 26, and 37% lower in phenolics than sample 1B, respectively. The amount of secoiridoids, expressed as the sum of total HYTY and TY, were in all samples higher than 5 mg per 20 g of oil as required for the application of the polyphenol health claim (EU Reg, 432/2012).

## 4 Conclusion

The Italian olive sector boasts a heritage of more than 400 cultivars representing the different territories and the highest number internationally of designations of origin. The latter must comply with product specifications/regulations and must be easily recognizable and distinguishable from other conventional products for consumers, promoting quality and authenticity. It is important to investigate the relationships between the quality of the raw material (olives) and those of the final product (EVOO) with a specific and in-depth focus on monovarietal oils. With this in mind, a new quality protocol aimed at maximizing the content in bioactive minor compounds of the monovarietal *Nostrana di Brisighella* olive oil can be developed aimed especially at protecting traditional and high value-added agri-food products (e.g., PDO, PGI, organic). For the latter, in fact, it may be crucial to determine which cultivars may be more suitable for ongoing climate change in relation to the agronomic system adopted. Indeed, this experimental work focuses on the study of the volatile compounds, the phenolic fraction, and sensory attributes of samples obtained from olives of *Nostrana di Brisighella* cv. produced with different agronomical practices to maximize the content of minor compounds in the EVOO produced. The MI of olives obtained through integrated pest management and organic farming may influence the quality indices of EVOO. From the results obtained, an increase in MI that followed the trend of the weeks of harvest was observed, and differences in the composition of the volatile and phenolic profile were highlighted that could also depend on the agronomic systems used. From sensory analysis, the “fruity,” “bitter,” and “pungent” sensory notes tended to have a higher intensity in the EVOOs produced in the first week of harvest, and these attributes decreased variably in samples obtained from riper olives in accordance with the trend seen for volatile C_5_ compounds. A decrease in phenolic compounds, as the ripening of olives progresses (in relation to the MI), was observed in the four weeks considered, with a clear decrease at more advanced veraison. A significant difference was also highlighted in relation to the agronomic system applied as the samples obtained from integrated pest management were richer in phenolic compounds overall than those derived from organic farming. Furthermore, all samples analyzed showed a phenolic content between 10.8 and 6.8 mg of HYTY and TY (free and bound forms), which is far above the 5 mg per 20 g of oil required for the application of the polyphenol health claim in the olive oil label. Regarding this last point, it will be important to investigate whether this concentration is preserved during storage. In this regard, the present work lays the foundation for the creation of a database regarding the content in minor compounds, specifically in relation to phenolic compounds, and constitutes the starting point for an ongoing study of the shelf-life of the same EVOOs analyzed herein.

## Data availability statement

The raw data supporting the conclusions of this article will be made available by the authors, without undue reservation.

## Author contributions

EC: Conceptualization, Data curation, Formal analysis, Investigation, Methodology, Writing – original draft, Writing – review & editing. EV: Conceptualization, Investigation, Supervision, Writing – review & editing. AB: Conceptualization, Data curation, Investigation, Methodology, Supervision, Writing – review & editing. SB: Data curation, Formal analysis, Writing – review & editing. RT: Formal analysis, Writing – original draft, Writing – review & editing. FF: Data curation, Formal analysis, Investigation, Methodology, Writing – original draft, Writing – review & editing. TT: Conceptualization, Funding acquisition, Supervision, Writing – review & editing.
